# The Development of Symbolic and Non-Symbolic Number Line Estimations: Three Developmental Accounts Contrasted Within Cross-Sectional and Longitudinal Data

**DOI:** 10.5334/pb.276

**Published:** 2016-12-20

**Authors:** Delphine Sasanguie, Lieven Verschaffel, Bert Reynvoet, Koen Luwel

**Affiliations:** 1 Brain and Cognition, KU Leuven, 3000 Leuven, Belgium; 2 Faculty of Psychology and Educational Sciences@Kulak, KU Leuven Kulak, 8500 Kortrijk, Belgium; 3 Centre for Instructional Psychology and Technology, Research Unit Education and Training, KU Leuven, 3000 Leuven, Belgium; 4 Centre for Educational Research and Development, KU Leuven – Campus Brussel, 1000 Brussel, Belgium

**Keywords:** cognitive development, numerical cognition, number line estimation, log-to-linear account, twolin-to-lin transformation, proportion judgment account

## Abstract

Three theoretical accounts have been put forward for the development of children’s response patterns on number line estimation tasks: the log-to-linear representational shift, the two-linear-to-linear transformation and the proportion judgment account. These three accounts have not been contrasted, however, within one study, using one single criterion to determine which model provides the best fit. The present study contrasted these three accounts by examining first, second and sixth graders with a symbolic and non-symbolic number line estimation task (Experiment 1). In addition, first and second graders were tested again one year later (Experiment 2). In case of symbolic estimations, the proportion judgment account described the data best. Most young children’s non-symbolic estimation patterns were best described by a logarithmic model (within the log-to-lin account), whereas those of most older children were best described by the simple power model (within the proportion judgment account).

## Introduction

In the past decade, mental representations of numbers and their development have been investigated intensively (e.g. Defever, Sasanguie, Vandewaetere & Reynvoet, 2012; Kucian & Kaufman, 2009; Reynvoet, De Smedt & Van den Bussche, 2009; Siegler & Opfer, 2003). It is commonly assumed that numbers are mentally represented akin to a ‘mental number line’, on which each number is represented as a Gaussian distribution around the corresponding mental magnitude (Dehaene, 1997). Moreover, these representations are assumed to obey Weber-Fechner’s law (Fechner, 1860), referring to larger overlapping Gaussian distributions with increasing magnitude. These mental representations allow people to determine magnitudes in an approximate way and therefore have been referred to as the ‘Approximate Number System’ (ANS; Barth, Beckmann & Spelke, 2008; Halberda & Feigenson, 2008). Also symbolic skills that are typically taught in school are hypothesized to be fostered by this pre-existing non-symbolic number system (Mundy & Gilmore, 2009; but see Noël & Rousselle, 2011; Sasanguie, Defever, Maertens & Reynvoet, 2013 for an alternative view). A widely used task to investigate how people represent numbers, is the *number line estimation task* (e.g. Berteletti, Lucangeli, Piazza, Dehaene & Zorzi, 2010; Dehaene, Izard, Spelke & Pica, 2008; Sasanguie, De Smedt, Defever & Reynvoet, 2012; Siegler & Opfer, 2003). In this task, participants are typically asked to place a given number on an empty number line which is bounded by a starting value, usually zero or one, at the beginning of the line, and another value, such as 100 or 1000, at the end of the line. These numbers can be either symbolic (e.g. Arabic digits) or non-symbolic (e.g. dot patterns).

**Figure 1:**
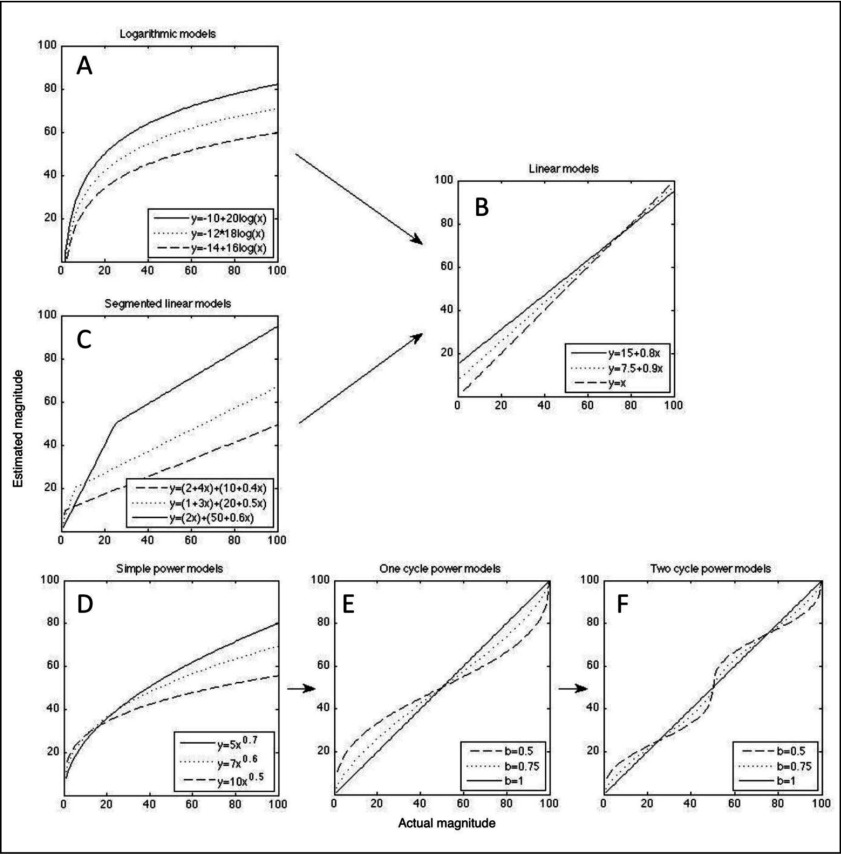
Predicted estimation data for the three developmental accounts of number line estimation: the log-to-lin account **(A-B),** the twolin-to-linear account **(C-B)** and the proportion judgment account, consisting of a transformation of a power model **(D),** over a one-cycle power model **(E),** to a two-cycle power model **(F)**. Each model is presented with a combination of three parameters.

Siegler and Opfer (2003) suggested that the underlying numerical magnitude representations can be derived from number line estimation tasks by regressing the actual magnitudes (x) on the estimated magnitudes (y). Doing so, researchers have shown that, with increasing age, children’s estimations on a symbolic number line (e.g. Booth & Siegler, 2006; 2008) and on a non-symbolic number line (e.g. Sasanguie et al., 2012; Sasanguie, Göbel, Moll, Smets & Reynvoet, 2013) evolve from a logarithmic (i.e. with smaller magnitudes being overestimated and larger magnitudes being underestimated, see Figure 1A), to a more precise, linear pattern (see Figure 1B). Moreover, it has been shown that this so-called *logarithmic-to-linear (log-to-lin) shift* is dependent on the range of the number line and participants’ age: between kindergarten and second grade, children make the log-to-lin shift on a 0–100 number line, between second and fourth grade this occurs for the 0–1000 number line and between third and sixth grade, children shift towards a linear representation on a 0–100 000 number line (Siegler, Thompson & Opfer, 2009). Therefore, it is assumed that an increase in linearity – and thus more accurate estimations – are dependent on children’s familiarity with a certain number range (Siegler & Opfer, 2003).

In contrast with these studies that provided evidence for a log-to-linear representational shift, other researchers have put forward an alternative model for the development of number representations (e.g. Ebersbach, Luwel, Frick, Onghena & Verschaffel, 2008; Moeller, Pixner, Kaufmann & Nuerk, 2009): a representational shift from a *two-linear or two-phase segmented model* (see Figure 1C) to a simple linear model (twolin-to-lin account, see Figure 1B). The two-linear model consists of two separate linear models with a steep slope for small magnitudes and a shallow slope for larger magnitudes. Considering the breakpoint between the two linear segments, there is, however, no consensus: Whereas Ebersbach et al. (2008) suggest that the breakpoint is variable and characterizes the end of the number range children are familiar with, Moeller et al. (2009) believe that the breakpoint is fixed and represents the switch between one- and two-digit numbers. Either way, the segmented linear model has been found to describe the number line estimation performance of children better than the logarithmic or the linear model, as evidenced by a larger mean coefficient of determination adjusted for the amount of parameters (i.e. adjusted *R^2^*; Ebersbach et al., 2008; Moeller et al., 2009).

More recently, a third developmental account has been proposed: *the proportion judgment account* (Barth & Paladino, 2011). Here, it is assumed that participants solve the number line task by estimating the target value as a proportion of the total length of the number line. Initially, participants estimate magnitudes only by relying on the begin point or using a wrong value as the endpoint of the number line. This results in a *simple power model*^1^ that can explain the relationship between the actual and the estimated magnitudes (see Figure 1D). Later on, participants start to estimate magnitudes based on the total proportion of the number line, using both the begin- and endpoint. A *one-cycle power model* is needed to explain the data: a combination of two power models meeting in the midpoint of the number line (see Figure 1E). Estimations will consequently be more accurate around the midpoint (e.g. 50 in a 0–100 line), whereas magnitudes below the midpoint will be overestimated and magnitudes above will be underestimated. Finally, participants will, next to the begin- and endpoint, also use an intermediate, internal reference point at the middle of the number line. In this case, the data can be modelled by a *two-cycle power model* (see Figure 1F). This model is a combination of four power functions that meet in the quartiles of the scale of the number line. Two cycles of accurate estimations at the quartiles (e.g. 25 and 75 in a 0–100 line) of the number line, and an overestimation for numbers below 25 and between 50 and 75, in combination with an underestimation for numbers between 25 and 50 and above 75 is expected. In sum, the three-step transformation from a simple power model, over a one-cycle power model to a two-cycle power model is considered to be the result of a gradual decrease of the bias of estimations (parameter b) and an increase of the number of reference points used over development.

Despite the ongoing debate (e.g. Ashcraft & Moore, 2012; Barth & Paladino, 2011; Barth, Slusser, Cohen & Paladino, 2011; Ebersbach et al., 2008; Moeller et al., 2009; Opfer, Siegler & Young, 2011; Slusser, Santiago & Barth, 2013; White & Szücs, 2012; Xu, Chen, Pan & Li, 2013; Young & Opfer, 2011), it remains to date unclear which of these three developmental accounts reflects best how children’s number line estimation patterns evolve through development. Slusser et al. (2013), for example, compared the fit of the three proportion judgment models with the fit of the logarithmic and the linear model. They examined 5- through 10-year old children with symbolic number line estimation tasks within familiar (e.g. 0–20 and 0–100) and unfamiliar number ranges (e.g. 0–1000 and 0–10000), dependent on the age of the children. Both the median best fitting model and the best fitting model per individual were calculated, based on the ‘Akaike Information Criterion corrected for small samples’ (AICc; Burnham & Anderson, 2004). Results revealed for both group and individual analyses that the proportion judgment account provided the best explanation of the observed estimation patterns.

In contrast, Ashcraft and Moore (2012) examined elementary school children and adults using a symbolic (0–100 and 0–1000) Position-to-Number (P-N) task (i.e. a variant of the number line estimation task in which participants have to estimate the number that corresponds with a given position on a number line) to determine the best fitting model by registering the highest coefficient of determination (*R^2^*) per individual of the exponential^2^, linear, one-cycle and two-cycle power model fitted on that individuals’ estimation pattern. They observed estimation patterns largely consistent with the log-to-lin shift account.

Another attempt to contrast the log-to-lin account with the proportion judgment account came from White and Szücs (2012). Here, children from grade 1–3 were presented with a symbolic 0–20 number line task and data were analysed both on a group and an individual level. Results also favoured the log-to-lin representational development, but at the same time pointed to the use of clever strategies that might underpin the development from a logarithmic to a linear magnitude representation. However, in this study, data of the cyclic power models (i.e. one-cycle and two-cycle power model) were pooled together when comparing them to the log and lin models and, as in Ashcraft and Moore (2012), the coefficient of determination (*R^2^*) was not corrected for the number of parameters in the models.

Finally, recently Xu, Chen, Pan and Li (2013) investigated the development of mental number representations in Chinese preschoolers. They compared not only the fit of the models from the log-to-lin account with those from the proportion judgment account, but also investigated the two-linear-to-linear transformation account in 5–6 year old children, using symbolic number lines (Arabic digits from 0–100 and 0–1000). Results showed that the estimates of these Chinese 5–6 year olds fitted the two-linear and the linear models better than the logarithmic, one-cycle and two-cycle power models. The simple power model from the proportion judgment account was however not included in the analyses. Moreover, the adjusted *R^2^* was used again instead of the more reliable AICc measure.

In sum, previous studies that attempted to shed light on which model fits children’s numerical estimation patterns best, have provided inconsistent results. The latter might be due to differences in age groups, type of task (i.e. N-P or P-N) and number line scales being investigated. Furthermore, different criteria have been used to determine which model provides the best fit with the data, such as *R^2^*, adjusted *R^2^*, and AICc. Finally, none of these studies has contrasted all models from each of the three developmental accounts. In order to provide a more complete picture of which developmental account characterizes best the development of children’s number line estimation patterns, we contrasted in the current study the three aforementioned developmental accounts (i.e. log-to-lin, two-lin-to-lin, and proportion judgment), using one scale (0–100), one type of task (N-P task) and the same single criterion (AICc). All statistical models involved in these three accounts (i.e. logarithmic, two-linear, linear, simple power, one-cycle and two-cycle power model) were investigated. Second, data were gathered not only for symbolic (Arabic digits) but also for non-symbolic (dot patterns) stimuli to test the extent to which the developmental trajectory for non-symbolic number line estimation mirrors the one of symbolic estimation described in the literature. Indeed, evidence on non-symbolic number lines is very limited: A log-to-lin shift has already been observed in adults (e.g. Anobile, Cicchini & Burr, 2012) and in children (e.g. Sasanguie et al., 2012; Sasanguie, Göbel et al., 2013), but the two other developmental accounts have not been investigated yet with non-symbolic stimuli. Third, to test the consistency in the observed developmental patterns, we investigated these developmental trajectories not only cross-sectionally (Experiment 1), but also longitudinally (Experiment 2). To the best of our knowledge, this is the first study that compares all three accounts with both symbolic and non-symbolic stimuli in a cross-sectional as well as a longitudinal design.

## Experiment 1: Cross-sectional study

### Method

#### Participants

One-hundred and ten typically developing children from an elementary school in Flanders (Belgium) participated in this study: 33 first graders (*M*_age_ = 6.65 years, *SD* = .28, 13 males), 37 second graders (*M*_age_ = 7.60 years, *SD* = .27, 16 males) and 40 sixth graders (*M*_age_ = 11.62 years, *SD* = .36, 15 males). All children participated in the symbolic and the non-symbolic number line task. First and second graders were considered as crucial for this study, because of their well-documented logarithmic-to-linear shift at that age (Booth & Siegler, 2006; Sasanguie et al., 2012; Siegler & Booth, 2004) and their twolinear-to-linear shift in the 0–100 range (e.g. Xu et al., 2013). Sixth graders were included to shed light on the estimation patterns of older children, to obtain a more complete picture of children’s developmental trajectory.

#### Materials and procedure

The number line estimation tasks were presented to the children on white A4 sheets. The general outline of the stimulus material was consistent with the setup of Siegler and Opfer (2003). Number lines ranged from 0 to 100, both in the symbolic and the non-symbolic condition. Symbolic stimuli were Arabic digits (Arial font, size 16). Non-symbolic stimuli were white-filled circles (radius: 3.5cm) containing a set of black dots, which were generated with the MatLab script of Dehaene, Izard and Piazza (2005), controlling for item size and total occupied area. The end points of the number lines were labelled on the left by 0 and on the right by 100 in the symbolic condition and by an empty circle on the left and a circle with 100 dots on the right in the non-symbolic condition. Each line was 25cm long and was centred on the paper with the numerical magnitude that had to be positioned on the number line being presented 6 cm above the number line. The numerical magnitudes that had to be positioned on the number line were 2, 3, 4, 6, 18, 25, 48, 67, 71, 86 (corresponding to sets A and B for the same interval used in Siegler & Opfer, 2003). The presentation order of the magnitude was randomized and each line was presented on a separate sheet. Children were instructed to mark on the line were they thought that the magnitude had to be positioned. To ensure that children were aware of the interval size, the experimenter showed the first item of the task while saying: “This line goes from 0 (dots) to 100 (dots). If here is 0 and here is 100, where would you position this number (magnitude)?”. After that, the children were able to go through all sheets at their own pace.

### Data-analysis and results

Because using mean or median estimation data of an age group can obscure individual differences in the estimation patterns and trajectories, the fit of the models was based on individual estimation patterns instead of on aggregated data. For each model within each of the three developmental transformation accounts, we calculated the AICc measure (for a similar method, see Barth & Paladino, 2011; Burnham, Anderson & Huyvaert, 2011; Huber, Moeller & Nuerk, 2013; Slusser et al., 2013). In contrast to other measures (e.g. *R^2^*), AICc takes into account both goodness of fit and model complexity, where model complexity is defined in terms of the number of parameters (Burnham & Anderson, 2002). The AICc is calculated according to the following formula:

\[
AICc = - 2\ln \left({\frac{{RSS}}{n}} \right) + 2K + \frac{{2K\left({K + 1} \right)}}{{n - K - 1}}
\]

where the *RSS* is the residual sum of squares, *n* is the number of data samples, and *K* is the number of predictors of the model. As recommended by Burnham and Anderson (2002; see also Slusser et al., 2013), models were ranked on the basis of ΔAICc. This measure refers to the differences in AICc between the “best” model (i.e. the model with the lowest AICc) and the AICc of the other models involved in the comparison. For example, if one wants to determine whether the logarithmic model with an AICc of 55 or the linear model with an AICc of 43 describes the data of a particular child best, one has to calculate the difference in AICc with the best model. In the present case, the linear model would be the best and the logarithmic model would have a ΔAICc of 12 (= 55 – 43). Burnham and Anderson (2002) provide a rule of thumb which states that models having a ΔAICc within 0–2 of the best model have substantial support and should be taken into consideration when making inferences, models with a ΔAICc within 4–7 have considerably less support and models with a ΔAICc >10 have essentially no support. Application of this rule of thumb to the above example would indicate that the linear model would describe the data much better than the logarithmic model which has essentially no support (ΔAICc = 12).

Following Slusser et al. (2013), participants were excluded from the analyses if they did not meet one of the following criteria: First, each participant had to exhibit a significantly positive correlation between the estimated and actual magnitudes. Second, participants who estimated 90% of the stimuli within less than 10% of the number line were excluded. Third, to ensure that the overall best fitting model could be reliably determined by means of the AICc, we also excluded participants whose residual sum of squares of a particular model deviated more than three standard deviations from the mean residual sum of squares of that model.

We first discuss the results of the symbolic task and afterwards those of the non-symbolic task. For both tasks, error rates of the different age groups are compared. Next, to determine the percentage of participants per grade that were best fitted by a particular model, the frequencies of the best fitting models within each developmental account are discussed for the different age groups. Finally, we report the results of the analysis of which developmental account provides the best fit.

#### Symbolic number line task

Based on the above-mentioned criteria, three first graders and one second grader were removed from the data set. Accordingly, the final sample for the analyses on the symbolic number line data consisted of 30 first, 36 second, and 40 sixth graders.

##### Error rates

Participants’ error rates were measured in terms of the mean percent absolute error (PAE), using the formula of Siegler and Booth (2004):

\[
\left. {\left| {\frac{{Estimate - Estimated\ \ {\rm{ }}Quantity}}{{Scale{\rm{ }}\ \ of\ \ {\rm{ }}Estimates}}} \right.} \right|
\]

In order to examine whether the performance on this task differed between grades, a one-way ANOVA was conducted on the PAEs. A significant main effect of grade was observed, *F*(2, 103) = 50.66, *p* < .001, η*_p_^2^* = .50, indicating an increase in accuracy with grade (see Table 1). Tukey post-hoc tests revealed significant differences between all grades, all *p*s ≤ .005.

**Table 1:** Mean percentages of absolute error (PAE) (and the corresponding standard deviations) on the symbolic and the non-symbolic number line estimation tasks, per grade.

Grade	Mean PAE	
	*Symbolic number line estimation task*	*Non-symbolic number line estimation task*

**1^st^ grade**	11.48 *(4.93)*	19.46 *(5.48)*
**2^nd^ grade**	7.83 *(3.83)*	13.52 *(4.82)*
**6^th^ grade**	3.08 *(1.04)*	8.36 *(3.13)*

##### Frequency analysis on the best fitting models within each developmental account

In line with the three above-mentioned developmental accounts, three different model comparisons were carried out for each participant: (1) a comparison of a logarithmic with a linear model for the log-to-lin shift account, (2) a comparison of a two-linear with a simple linear model for the two-lin-to-lin transformation account and (3) a comparison of a simple power model with a *n*-cycle power model for the proportion judgment account^3^. Table 2 shows, for each grade and developmental account, the percentage of participants whose estimation pattern was best described by a particular model, accompanied by the mean ΔAICc. We performed a Chi-square analysis to examine whether there were significant changes between the different grades with respect to the number of children whose estimation pattern was best described by a particular model. For the log-to-lin account, the expected developmental model transformation from log-to-lin was confirmed by a significant association between the best model and grade, *χ²*(2) = 34.02, *p* < .0001: The estimation pattern of most first graders (i.e. 63%) was best described by the logarithmic model. However, with increasing grade, there was an increase in the percentage of children having an estimation pattern being best described by the linear model. Table 2 shows that the mean ΔAICcs accompanying the linear model (i.e. the difference in AICc between the logarithmic model and this “best”, linear model) increased with grade from 11.82 to 27.29, demonstrating increasingly less support for the logarithmic model in each of the model comparisons. Also the developmental twolin-to-lin model transformation was confirmed by a significant association between the best model and grade, *χ²*(2) = 6.06, *p* < .05. The percentage of participants whose estimation pattern was best described by the linear model increased with increasing grade. Mean ΔAICcs for the linear model in this account also increased with grade from 4.71 to 6.67, revealing increasingly less support for the two-linear model. For the proportion judgment account, the association of the best model and grade failed to reach significance, *χ²*(4) = 2.06, *p* = .36. However, as can be derived from Table 2, the percentage of participants whose estimation pattern was best fitted by a *n*-cycle power model tended to increase from first to sixth grade. Mean ΔAICcs for the *n*-cycle power model increased slightly from 3.20 to 4.32, indicating a slight decrease in support for the simple power model.

**Table 2:** Percentage of children whose symbolic number line estimation pattern is best described by a specific model and the corresponding mean ΔAICc (SD in parentheses), for each developmental account and per grade.

	Grade					
	1		2		6	
	
Model	% children	Mean ΔAICc	% children	Mean ΔAICc	% children	Mean ΔAICc

Log-Lin Account						
Log	63	7.74 (4.15)	31	7.20 (4.15)	0	–
Lin	37	11.82 (7.49)	69	13.94 (7.49)	100	27.29 (7.58)
	
2Lin-Lin Account						
2Lin	37	5.77 (4.06)	19	6.68 (4.86)	13	4.42 (3.65)
Lin	63	4.71 (2.21)	81	6.02 (2.74)	87	6.67 (2.02)
	
Proportion Judgement Account						
Pow	47	5.23 (6.52)	36	4.13 (5.17)	30	4.64 (5.38)
*n* -Cycle	53	3.20 (2.45)	64	4.20 (3.65)	70	4.32 (3.10)

*Note*. ΔAICc is the difference in Aikaike’s Information Criterium corrected for small samples between the best model and the other model(s) in the same developmental account.

##### Developmental account best reflecting the development of estimation patterns

The previous analysis determined for each child the *model* describing the data best (i.e. the “best” model) within each of the three developmental accounts. In the present analysis, we compared, for each child, the best model in each developmental account with the best model from the other two accounts on the basis of ΔAICc to determine which of the three *accounts* would describe children’s data best (see Table 3). As can be derived from the mean values, the estimation pattern of the majority of the children in each grade (i.e. 53%, 75% and 75% in first, second and sixth grade respectively) was best described by the proportion judgment account (see all mean ΔAICcs, reflecting strength of evidence in favour of this account > 3.30). A Chi-square analysis revealed a significant association between grade and account type, *χ²*(4) = 10.84, *p* = .03. The percentage of children for whom the proportion judgment account was the best account increased slightly with grade, while there was an opposite pattern of results with respect to the log-lin account. The mean ΔAICcs for this log-lin account were somewhat larger than for the proportion judgment account, except in sixth grade. The two-lin-to-lin account was the least preferred account in all grades and did not change strongly amongst grades.

**Table 3:** Percentage of children whose symbolic number line estimation pattern is best described by a specific account in comparison to another account with the corresponding mean ΔAICc (SD in parentheses), per grade.

	Log-Lin		2Lin-Lin		Prop		Mean	
	
Best Account	% children	MeanΔAICc	% children	Mean ΔAICc	% children	Mean ΔAICc	% children	Mean ΔAICc

1st Grade								
Log-Lin	–	–	40	6.02 (2.41)	40	3.61 (2.63)	40	4.81 (2.78)
2Lin-Lin	7	1.57 (1.34)	–	–	7	2.88 (2.25)	7	2.22 (1.69)
Prop	53	2.85 (2.39)	53	3.70 (2.35)	–	–	53	3.30 (2.38)

2nd Grade								
Log-Lin	–	–	17	6.51 (2.78)	25	2.90 (3.15)	21	4.35 (3.43)
2Lin-Lin	0	–	–	–	8	3.93 (0.78)	4	3.93 (0.78)
Prop	75	4.91 (3.61)	75	4.66 (2.78)	–	–	75	4.79 (3.19)

6th Grade								
Log-Lin	–	–	0	–	20	0.81 (0.88)	10	0.81 (0.88)
2Lin-Lin	5	5.55 (6.09)	–	–	25	1.08 (0.96)	15	2.11 (2.87)
Prop	75	4.04 (2.86)	75	3.67 (2.69)	–	–	75	3.85 (2.92)

*Note*. AICc = Aikaike’s Information Criterium corrected for small samples. The rows display the percentage of children whose estimation pattern is best described by a specific account, together with the mean ΔAICc, reflecting the strength of evidence in favour of this account compared to each of the other two accounts (columns), respectively.

#### Non-symbolic number line task

The same three exclusion criteria as in the symbolic number line condition were applied, resulting in the exclusion of one first grader, three second graders, and one sixth grader from the data analyses on the non-symbolic number line task. Accordingly, the final sample here consisted of 32 first, 34 second, and 39 sixth graders.

##### Error rates

A one-way ANOVA on the mean PAEs revealed a significant main effect of grade, *F*(2,102) = 53.35, *p* < .0001, η*_p_^2^* = .51, indicating increasing accuracies with grade (see Table 1). Tukey post-hoc tests revealed significant differences between all grades, all *p*s ≤ .001.

##### Frequency analysis on the best fitting models within each developmental account

Table 4 shows, for each grade and developmental account, the percentage of participants whose estimation pattern was best described by a particular model, together with the mean ΔAICcs. The log-to-lin transformation account was confirmed by a significant association between the best model and grade, *χ²*(2) = 21.34, *p* < .0001. With increasing grade, the percentage of participants whose estimation pattern was best described by the log model decreased, thus leading to more estimation patterns being best described by the linear model. Mean ΔAICcs for the linear model increased with age from 6.35 to 12.95, indicating increasingly less support for the logarithmic model. The developmental transformation from a two-linear to a linear model was also confirmed by a significant association of the best fitting model and grade, χ²(2) = 10.72, *p =* .005. With increasing grade, there was an increase in the percentage of participants whose estimation pattern was best described by the linear model. Mean ΔAICc for the linear model decreased slightly from first (i.e. 3.16) to second grade (i.e. 2.44) but then increased strongly to 7.12, revealing less support for the two-linear model. Similar to the symbolic number line data, we did not observe an association between the different proportion models and grade, χ²(2) = 2.88, *p* = .33. However, the data in Table 4 suggest that the number of participants whose estimation pattern was best described by a *n*-cycle power model increased with age. Mean ΔAICc values ranged for the *n*-cycle model between 3.13 and 4.14, indicating again considerably less support for the simple power model.

**Table 4:** Percentage of children whose non-symbolic number line estimation pattern is best described by a specific model and the corresponding mean ΔAICc (SD in parentheses), for each developmental account and per grade.

	Grade					
	1		2		6	
	
Model	% children	Mean ΔAICc	% children	Mean ΔAICc	% children	Mean ΔAICc

Log-Lin Account						
Log	78	11.80 (4.51)	48	6.48 (4.16)	23	5.49 (4.12)
Lin	22	6.35 (6.65)	52	8.80 (6.57)	67	12.95 (9.91)
	
2Lin-Lin Account						
2Lin	72	12.10 (8.15)	56	8.19 (6.72)	33	5.14 (3.90)
Lin	28	3.16 (2.47)	44	2.44 (1.14)	67	7.12 (2.26)
	
Proportion Judgement Account						
Pow	88	10.15 (4.64)	82	7.72 (5.76)	72	7.90 (5.73)
*n*-Cycle	12	3.13 (1.53)	18	4.14 (3.80)	28	3.46 (3.64)

*Note*. ΔAICc is the difference in Aikaike’s Information Criterium corrected for small samples between the best model and the other model(s) in the same developmental account.

##### Developmental account best reflecting the development of estimation patterns

Table 5 shows the percentage of children whose estimation pattern is best described by a specific account, together with the mean ΔAICc. A Chi-square analysis revealed a marginally significant association between grade and account, *χ²*(4) = 8.77, *p* = .07. As for the symbolic number line data, we observed a trend wherein the percentage of children for whom the proportion judgment account was the preferred account increased with grade, while there was an opposite pattern of results with respect to the log-lin account. Again, the two-lin-to-lin account did not exhibit a consistent pattern of change. Mean ΔAICc values ranged between 2.59 and 9.48, suggesting in most cases considerable support for the best account.

**Table 5:** Percentage of children whose non-symbolic number line estimation pattern is best described by a specific account in comparison to another account, with the corresponding mean ΔAICc (SD in parentheses), per grade.

	Log-Lin		2Lin-Lin		Prop		Mean	
	
Best Account	% children	Mean ΔAICc	% children	Mean ΔAICc	% children	Mean ΔAICc	% children	Mean ΔAICc

1st Grade								
Log-Lin	–	–	41	4.33 (2.71)	41	5.32 (2.87)	41	4.83 (2.78)
2Lin-Lin	28	5.75 (6.00)	–	–	28	13.21 (6.48)	28	9.48 (7.17)
Prop	31	3.12 (2.27)	31	4.32 (3.28)	–	–	31	3.72 (2.81)

2nd Grade								
Log-Lin	–	–	15	3.67 (2.64)	32	2.36 (2.20)	24	2.59 (2.30)
2Lin-Lin	24	4.09 (3.17)	–	–	47	4.40 (5.17)	35	4.30 (4.53)
Prop	41	5.31 (4.36)	38	3.90 (2.46)	–	–	41	4.63 (3.58)

6th Grade								
Log-Lin	–	–	8	5.36 (1.70)	28	1.62 (2.30)	18	3.49 (2.65)
2Lin-Lin	10	6.16 (3.97)	–	–	31	2.39 (2.84)	21	3.33 (3.45)
Prop	62	3.67 (3.42)	62	3.30 (2.70)	–	–	62	3.49 (1.33)

*Note*. AICc = Aikaike’s Information Criterium corrected for small samples. The rows display the percentage of children whose estimation pattern is described best by a specific account, together with the mean ΔAICc, reflecting the strength of evidence in favour of this account compared to each of the other two accounts (columns), respectively.

### Discussion

The aim of the current study was to provide a complete picture of which developmental account (log-to-lin account, twolin-to-lin account or proportion judgment account) best characterizes the development of children’s symbolic and non-symbolic number line estimation patterns, by including all the models of each of the three developmental accounts into the analyses and using two complementary methodological approaches. In Experiment 1, we investigated this cross-sectionally by testing 1^st^, 2^nd^ and 6^th^ graders.

Results of Experiment 1 provided evidence for all three developmental accounts described in the literature (e.g. Barth & Paladino, 2011; Ebersbach et al., 2008; Siegler & Opfer, 2003). In line with the *log-to-lin transformation account*, the symbolic number line data suggested that, with increasing grade, the percentage of children exhibiting a logarithmic estimation pattern decreased, whereas the percentage of children who showed a linear estimation pattern increased. This trend is consistent with previous results (e.g. Aschraft & Moore, 2012; Booth & Siegler, 2008; Sasanguie et al., 2012). A similar pattern was observed in the non-symbolic task, albeit with a certain delay: Although the percentage of children exhibiting a linear estimation pattern increased, the percentage of children still showing a logarithmic estimation pattern was, in comparison with the symbolic condition, larger here (e.g. for the non-symbolic task, not all sixth graders exhibited a linear estimation pattern yet). The present log-to-lin representational shift for both symbolic and non-symbolic number line estimations, based on the AICc, is completely in line with previous findings by Sasanguie et al. (2012) who used *R^2^* as a criterion to determine the best model fit. In accordance with the *twolin-to-lin developmental account*, the symbolic number line data demonstrated that, with increasing age, children evolve from a two-linear to a simple linear estimation pattern. In case of the non-symbolic number line data, again a similar but delayed developmental transformation towards a linear estimation pattern was observed: The percentage of participants demonstrating a two-linear estimation pattern was in each age group considerably higher than in the symbolic condition. For the first graders, for example, the linear model best fitted twice as much estimation patterns for the symbolic than for the non-symbolic task. These non-symbolic data extend the findings of Ebersbach et al. (2008), who observed the same developmental transformation of children’s estimation performance on the 0–100 symbolic number line task. Finally, the *proportion judgment account* was reflected in both the symbolic and non-symbolic data by a trend towards a decreasing number of children exhibiting an estimation pattern being best fit by a simple power model with increasing grade. As in the two previous developmental accounts, the proportion judgment account also showed a developmental delay in the non-symbolic compared to the symbolic data: More children exhibited an estimation pattern being best fit by a simple power model in all age groups for the non-symbolic task in comparison to the symbolic task. Again, these symbolic data confirm and the non-symbolic data extend the previously observed estimation patterns by Barth and colleagues (Barth & Paladino, 2011; Slusser et al., 2013).

When comparing several developmental accounts, in case of the *symbolic number line estimation task*, our findings revealed that the proportion judgment account best reflected the development of symbolic number line estimation patterns in all grades. These findings are highly similar to what Slusser et al. (2013) demonstrated, except that these researchers observed that in case of the first graders, the log-to-lin account and the proportion judgment account described the data equally well, while this was not the case in our data (see Table 3, mean ΔAICc = 2.85). The observation that especially the proportion judgment account (and the *n*-cycle power model within this account) best describes the estimation pattern of most children is in contrast with Xu et al. (2013) who observed a better fit for the two-linear and the linear model in 5–6 year old children than for the logarithmic or *n*-cycle power models. Moreover, this is in contrast with Ashcraft and Moore (2012), who observed a better fit for the log (exponential)-to-lin account than for the models of the proportion judgment account, in grades 1–5. However, in those studies, *R^2^* values instead of AICc values were used and the simple power model from the proportion judgment account was not considered in their analyses. Moreover, Ashcraft and Moore (2012) used a P-N task instead of a N-P task. These three differences might account for the contrasting results. Additional support for this claim is the aforementioned observation that our results are more in line with those of Slusser et al. (2013), who also made use of the AICc, a N-P task, and considered all models of the proportion judgment account into their analyses. For the *non-symbolic number line task*, we observed that the estimation patterns of the first graders were described better by the log-to-lin account, whereas for the estimation patterns of the second- and the sixth graders again the proportion judgment account best reflected the development. To date, no study exists in which the three developmental accounts have been contrasted using non-symbolic number line estimation data. Therefore, we cannot discuss this finding with respect to previous studies. However, we elaborate more on this finding in the general discussion. Finally, it should be noted that the ΔAICc values were somewhat smaller when comparing models *between* accounts than when comparing models *within* a particular account. A plausible explanation would be that, in the comparisons *between* the accounts, the best fitting models from the respective accounts compete with each other, whereas in the comparisons *within* an account a worse and a best fitting model compete with each other.

To increase the robustness of these findings, a second experiment was conducted in which the development of children’s symbolic and non-symbolic estimation patterns were investigated by means of a longitudinal design. Such a longitudinal approach has already been followed by Geary et al. (2008) and Muldoon, Towse, Simms, Perra and Menzies (2013) for the log-to-lin account, but not for the other two accounts.

## Experiment 2: Longitudinal study

### Method

#### Participants

Participants were the first and second graders of Experiment 1 who were retested one year later. From three first graders and one second grader, data could not be obtained at this second test moment (T2). As a result, the re-tested sample consisted of 30 second graders (*M*_age_ = 95.07 months, *SD* = 3.39, 12 males) and 36 third graders (*M*_age_ = 106.49 months, *SD* = 3.39, 15 males). From now on, these two developmental groups will be referred to as Cohort 1 (i.e. group of first graders at T1) and Cohort 2 (i.e. group of second graders at T1).

#### Materials and procedure

The materials and the procedure for Experiment 2 were identical to Experiment 1.

### Data analysis and results

The longitudinal results for the symbolic number line task are reported first, followed by those for the non-symbolic task. First, error rates of the different age groups within each cohort are compared. Next, the percentages of children whose individual estimation pattern fits best with a particular model are discussed for each developmental account. Finally, we analyzed which developmental account provides the best fit in each of the two cohorts.

#### Symbolic number line task

Based on the same exclusion criteria as in Experiment 1, two children from Cohort 1 were excluded from the analysis. This resulted in a sample of 27 children in Cohort 1 and 32 in Cohort 2.

##### Error rates

We examined whether the accuracy on the number line task improved with grade by means of a *t*-test for dependent samples on the PAEs, for each cohort separately. For Cohort 1, we observed that the PAE in grade 1 (*M* = 11.21, *SD* = 5.02) was significantly larger than in grade 2 (*M* = 7.57, *SD* = 3.24), *t*(26) = 4.78, *p* < .0001. Similarly, in Cohort 2, the PAE in grade 2 (*M* = 7.92, *SD* = 3.92) was significantly larger than in grade 3 (*M* = 4.88, *SD* = 2.56), *t*(31) = 5.52, *p* < .0001.

##### Frequency of the best fitting models within each developmental account

Table 6 shows, for each cohort and developmental account, the percentage of participants whose estimation pattern was described by the ‘best model’ within the account, together with the mean ΔAICc. The longitudinal data in Cohort 1 showed the expected developmental trend for both the log-lin and the two-lin-to-lin account, indicating an increase in the percentage of children whose estimation pattern is best described by the most advanced model within these accounts, namely the linear model. For the proportion judgment account, there were no substantial changes from grade 1 to grade 2 in the percentage of children whose estimation pattern was described best by a specific model. Mean ΔAICc values were all larger than 3 (range: 3.94 – 20.30) for the log-lin account and larger than 4 for the two-lin-to-lin account (range: 4.83 – 11.80), indicating considerable support for the best model in each comparison. For the proportion judgment account, mean ΔAICc values lied between 3 and 5 (range: 3.20 – 4.32) reflecting somewhat less support for the best model in each comparison compared to the other two accounts. For Cohort 2, we observed for all accounts the expected developmental trend. As for Cohort 1, mean ΔAICc values were on overall largest for the log-to-lin account (range: 7.06 – 19.74), followed by the two-lin-to-lin account (range: 4.46 – 7.37) and the proportion judgment account (range: 3.97 – 7.77). So, all best models in all three accounts received considerable support.

**Table 6:** Percentage of children whose symbolic number line estimation pattern is best described by a specific model and the corresponding mean ΔAICc (SD in parentheses), for each developmental account, per grade and per cohort.

	Cohort 1			
	Grade 1		Grade 2	
	
Model	% children	Mean ΔAICc	% children	Mean ΔAICc

Log-Lin Account				
Log	60	6.80 (2.71)	30	3.94 (3.59)
Lin	40	11.82 (7.49)	70	20.30 (8.26)
	
2Lin-Lin Account				
2Lin	33	5.04 (3.54)	26	8.91 (7.02)
Lin	67	4.83 (2.11)	74	11.80 (8.52)
	
Proportion Judgement Account				
Pow	41	3.53 (3.10)	44	4.09 (3.41)
*n* -Cycle	59	3.20 (2.45)	56	4.32 (1.86)
**Model**	**Cohort 2**			

	**Grade 2**		**Grade 3**	
	
	**% children**	**Mean ΔAICc**	**% children**	**Mean ΔAICc**
	
Log-Lin Account				
Log	31	7.06 (5.60)	3	10.19 (–)
Lin	69	13.79 (7.02)	97	19.74 (9.75)
	
2Lin-Lin Account				
2Lin	19	7.37 (4.94)	9	4.46 (3.91)
Lin	81	5.89 (2.80)	91	5.69 (3.47)
	
Proportion Judgement Account				
Pow	41	4.13 (5.17)	28	7.74 (9.65)
*n* -Cycle	59	4.17 (3.89)	72	3.97 (3.51)

*Note*. ΔAICc is the difference in Aikaike’s Information Criterium corrected for small samples between the best model and the other model(s) in the same developmental account.

##### Developmental account best reflecting the development of estimation patterns

The percentage of children in Cohort 1 whose estimation pattern was best described in terms of the twolin-to-lin or proportion judgment account increased from Grade 1 to Grade 2, while there was an opposite pattern of results for the log-lin account (see Table 7). Mean ΔAICc values ranged between 2.42 and 4.42, indicating considerable support for the best models. For Cohort 2, we observed an increase in the percentage of children whose estimation pattern was best described in terms of the proportion judgment account and an opposite pattern of results for the log-to-lin account. There was hardly any change for the two-lin-to-lin account. Mean ΔAICc values ranged between 2.64 and 4.77, indicating considerable support for the best models.

**Table 7:** Percentage of children whose symbolic number line estimation pattern is best described by a specific account in comparison to another account with the corresponding mean ΔAICc (SD in parentheses), per grade and per cohort.

	Cohort 1							
	Log-Lin		2Lin-Lin		Prop		Mean	
	
Best Account	% children	Mean ΔAICc	% children	Mean ΔAICc	% children	Mean ΔAICc	% children	Mean ΔAICc

1st Grade								
Log-Lin	–	–	33	5.77 (2.76)	37	2.65 (1.70)	35	4.13 (2.72)
2Lin-Lin	7	1.57 (1.34)	–	–	4	4.47 (–)	6	2.53 (1.93)
Prop	59	2.85 (2.39)	59	3.76 (2.35)	–	–	59	3.30 (2.38)

2nd Grade								
Log-Lin	–	–	4	12.05 (–)	15	1.29 (0.65)	9	3.44 (4.85)
2Lin-Lin	19	10.31 (6.24)	–	–	30	5.30 (5.67)	24	7.23 (6.18)
Prop	67	3.91 (1.99)	67	4.40 (2.15)	–	–	67	4.15 (2.03)
	**Cohort 2**							
	**Log-Lin**		**2Lin-Lin**		**Prop**		**Mean**	
	
**Best Account**	**% children**	**Mean ΔAICc**	**% children**	**Mean ΔAICc**	**% children**	**Mean ΔAICc**	**% children**	**Mean ΔAICc**

2nd Grade								
Log-Lin	–	–	16	6.60 (3.10)	25	3.17 (3.25)	20	4.49 (3.52)
2Lin-Lin	0	–	–	–	25	3.17 (3.25)	11	3.17 (3.25)
Prop	69	4.91 (3.81)	69	4.63 (2.93)	–	–	69	4.77 (3.67)

3rd Grade								
Log-Lin	–	–	3	7.12 (–)	16	1.74 (1.92)	9	2.64 (2.79)
2Lin-Lin	3	8.87 (–)	–	–	16	2.06 (1.89)	9	3.20 (3.25)
Prop	82	4.61 (2.37)	82	4.55 (2.35)	–	–	82	4.60 (2.39)

*Note*. AICc = Aikaike’s Information Criterium corrected for small samples. The rows display the percentage of children whose estimation pattern is described best by a specific account, together with the mean ΔAICc, reflecting the strength of evidence in favour of this account compared to each of the other two accounts (columns), respectively.

#### Non-symbolic number line task

Based on the aforementioned criteria, one child from Cohort 2 was excluded from the data analysis, resulting in a sample of 29 children in Cohort 1 and 31 children in Cohort 2.

##### Error rates

As for the symbolic number line data, we examined whether the accuracy of the estimations improved with grade by conducting, for each cohort separately, a *t*-test for dependent samples on the PAEs. For Cohort 1, we observed that the PAE in grade 1 (*M* = 19.02, *SD* = 5.58) was significantly larger than in grade 2 (*M* = 13.74, *SD* = 4.72), *t*(28) = 3.64, *p* = .001. Similarly, in Cohort 2, the PAE in grade 2 (*M* = 13.13, *SD* = 4.43) was significantly larger than in grade 3 (*M* = 10.05, *SD* = 4.81), *t*(29) = 3.39, *p* = .002.

##### Frequency of the best fitting models within each developmental account

Table 8 shows the percentage of children whose individual estimation pattern was best described by a particular model in each developmental account. The longitudinal data showed for both cohorts an increase in the percentage of children that were best described by the more advanced model for the log-to-lin account and the twolin-to-lin account, whereas such an increase was not observed for the proportion judgment account. Despite the developmental trend from the less advanced to the more advanced model within an account however, Table 8 also demonstrated that the non-symbolic estimation pattern of a considerable percentage of children, within each developmental account, was described best by the less advanced model. Mean ΔAICc values in Cohort 1 were all larger than 6 (range: 6.35 – 11.39) for the log-to-lin account, larger than 3 for the twolin-to-lin account (range: 3.16 – 12.04) and larger than 3 (range: 3.13 – 11.42) for the proportion judgment account, indicating considerable support for the best model in each comparison. A similar pattern was observed for Cohort 2, except for the log-lin account, where an equal number of second graders’ estimation pattern was best described by the logarithmic or the linear model. The mean ΔAICc values in this account provided considerable support for the best model and were higher for the linear (range: 9.28 – 12.03) than for the logarithmic model (6.14 – 6.28). Mean ΔAICc values for the more advanced models in the two other accounts ranged between 2.33 and 7.69, revealing in most cases considerable support for the best model.

**Table 8:** Percentage of children whose non-symbolic number line estimation pattern is best described by a specific model with the corresponding mean ΔAICc (SD in parentheses), for each developmental account, per grade and per cohort.

	Cohort 1			
	Grade 1		Grade 2	
	
Model	% children	Mean ΔAICc	% children	Mean ΔAICc

Log-Lin Account				
Log	76	11.39 (4.51)	45	7.58 (4.57)
Lin	24	6.35 (6.65)	55	8.11 (5.95)
	
2Lin-Lin Account				
2Lin	69	12.04 (8.33)	52	7.64 (5.62)
Lin	31	3.16 (2.47)	48	5.73 (3.17)
	
Proportion Judgement Account				
Pow	86	10.00 (4.84)	83	11.42 (5.95)
*n* -Cycle	14	3.13(1.53)	17	3.88 (2.67)
	**Cohort 2**			
	**Grade 2**		**Grade 3**	
	
**Model**	**% children**	**Mean ΔAICc**	**% children**	**Mean ΔAICc**

Log-Lin Account				
Log	50	6.14 (4.06)	32	6.28 (4.62)
Lin	50	9.28 (6.68)	68	12.03 (7.20)
	
2Lin-Lin Account				
2Lin	58	7.62 (6.44)	48	6.28 (5.58)
Lin	42	2.37 (1.44)	62	5.50 (3.41)
	
Proportion Judgement Account				
Pow	87	7.60 (5.84)	84	7.69 (4.87)
*n* -Cycle	13	4.96 (4.62)	16	2.33 (1.54)

*Note*. ΔAICc is the difference in Aikaike’s Information Criterium corrected for small samples between the best model and the other model(s) in the same developmental account.

##### Developmental account best reflecting the development of estimation patterns

The longitudinal data indicate for Cohort 1 an increase in the percentage of children whose estimation pattern is best described by the proportion judgment account and a decrease in the percentage of children being best described by the log-to-lin account (see Table 9). About one fourth of the children kept on being best described by the two-lin-to-lin account. For Cohort 2, we observed a slight increase (from 41% up to 50%) in the percentage of children being best described by the proportion judgment account and a slight decrease in the percentage of children being described best by the two-lin-to-lin account (34% to 22%). The percentage of children being described best by the log-lin account remained around 25%. Mean ΔAICc values in Cohort 1 ranged between 2.90 and 9.95, providing support for the best model. These values ranged in Cohort 2 between 2.66 and 5.66. The log-lin account received the least support in this cohort.

**Table 9:** Percentage of children whose non-symbolic number line estimation pattern is best described by a specific account in comparison to another account with the corresponding mean ΔAICc (SD in parentheses), per grade and per cohort.

	Cohort 1							
	Log-Lin		2Lin-Lin		Prop		Mean	
	
Best Account	% children	Mean ΔAICc	% children	Mean ΔAICc	% children	Mean ΔAICc	% children	Mean ΔAICc

1st Grade								
Log-Lin	–	–	38	3.84 (2.55)	38	4.79 (2.50)	38	4.32 (2.51)
2Lin-Lin	24	6.62 (6.62)	–	–	28	12.86 (6.84)	28	9.95 (7.25)
Prop	34	3.12 (2.27)	34	4.32 (3.28)	–	–	34	3.72 (2.81)

2nd Grade								
Log-Lin	–	–	14	4.29 (2.81)	21	1.96 (1.75)	17	2.94 (2.41)
2Lin-Lin	21	4.76 (6.09)	–	–	28	4.06 (4.35)	24	4.36 (4.96)
Prop	59	3.14 (1.49)	59	2.87 (1.36)	–	–	59	3.01 (1.41)
	**Cohort 2**							
	**Log-Lin**		**2Lin-Lin**		**Prop**		**Mean**	
	
**Best Account**	**% children**	**Mean ΔAICc**	**% children**	**Mean ΔAICc**	**% children**	**Mean ΔAICc**	**% children**	**Mean ΔAICc**

2nd Grade								
Log-Lin	–	–	16	3.67 (2.64)	32	2.57 (2.20)	25	2.75 (2.32)
2Lin-Lin	23	3.72 (3.22)	–	–	45	4.17 (5.03)	34	4.02 (4.43)
Prop	42	5.48 (4.49)	39	3.97 (2.56)	–	–	41	4.46 (3.69)

3rd Grade								
Log-Lin	–	–	19	3.65 (0.96)	35	2.13 (2.20)	28	2.66 (1.97)
2Lin-Lin	13	8.65 (2.50)	–	–	29	4.33 (5.95)	22	5.66 (5.43)
Prop	52	4.24 (1.89)	48	3.22 (2.14)	–	–	50	3.67 (2.14)

*Note*. AICc = Aikaike’s Information Criterium corrected for small samples. The rows display the percentage of children whose estimation pattern is described best by a specific account, together with the mean ΔAICc, reflecting the strength of evidence in favour of this account compared to each of the other two accounts (columns), respectively.

### Discussion

Experiment 2 aimed at investigating the development of children’s symbolic and non-symbolic estimation patterns by means of a longitudinal design, to check whether these longitudinal patterns mirrored the findings of Experiment 1 and of previous cross-sectional studies. First, frequencies revealed that the longitudinal data almost perfectly mirrored the cross-sectional data, in both symbolic and non-symbolic estimations. Indeed, the longitudinal data again provided evidence for the three developmental accounts and Tables 6 and 8 clearly demonstrate that, with increasing age, children evolve from the less to the more advanced model within a specific developmental account. In particular, Cohort 1-children’s model fit at T2 was similar as Cohort 2-children’s model fit at T1, whereas the model fit of the Cohort 2-children at T2 mirrored the fit of the sixth graders observed in Experiment 1. In addition, as in Experiment 1, a delay in the development of non-symbolic estimation patterns in comparison with symbolic estimation patterns was observed. These findings extend the results of Geary et al. (2008) and Muldoon et al. (2012) – who already observed these longitudinal developmental trajectories for symbolic data and the log-to-lin account – with similar developmental findings for the non-symbolic data and the other two accounts.

Second, the analyses considering the ‘best developmental account’ demonstrated with regards to the *symbolic number line estimation data*, that the longitudinal data of Cohort 1 and 2 mirror the cross-sectional data of grades 1–2 and 2–6 described in Experiment 1, respectively. Indeed, for both Cohort 1 and 2 the percentage of children whose estimation pattern was best described by the proportion judgment account increased over time. Moreover, the longitudinal *non-symbolic number line estimation data* of Cohorts 1 and 2 were in line with the cross-sectional data of grades 1–2 and 2–6 respectively. Most children’s estimation patterns were at first best described by the log-lin account, but this decreased over time, in favour of an increasing percentage of children whose estimation pattern was best described by the proportion judgment account. A similar delay of non-symbolic data in comparison with the symbolic data as observed in the cross-sectional data of Experiment 1 was thus replicated with this longitudinal design.

## General discussion

How do children’s symbolic and non-symbolic number line estimation patterns develop with age and which developmental account described in the literature reflects this development best? The current study was the first to compare three different developmental accounts in 1^st^, 2^nd^ and 6^th^ grade children, using one scale (0–100), one type of task (N-P task) and one criterion for comparing the different model fits (AICc). In order to provide a clear answer to this research question, children were presented with both symbolic and non-symbolic number lines in a cross-sectional (Experiment 1) as well as a longitudinal study (Experiment 2).

First, we observed, for all three developmental accounts, that the longitudinal data nicely mirrored the cross-sectional data: With increasing age, children evolved within each developmental account from the less advanced (logarithmic, twolinear or simple power model) to the more advanced (linear or *n*-cycle) model. This finding was observed in symbolic, but also in non-symbolic estimations, although with a certain delay in the latter case. In the proportion judgment account, however, this evolution was less pronounced, in both the (symbolic as well as non-symbolic) cross-sectional and longitudinal data. These observations are in line with and extend the results of Sasanguie et al. (2012) who also observed these patterns for the log-to-lin account, but did not investigate the other two accounts.

Second, both the cross-sectional and the longitudinal data revealed that, in case of symbolic estimation, with increasing age, the (*n*-cycle power model within the) proportion judgment account described children’s estimation patterns best. These results are in line with, but, more importantly, also extend the cross-sectional findings reported by Slusser et al. (2013), who used a comparable analytical approach. This suggests that shifts in children’s symbolic number line estimations do not reflect a developmental change in their mental representations of number, but rather that children might start using certain strategies that are based on (internal) anchor points (Barth & Paladino, 2011; Cohen & Sarnecka, 2014; Link, Huber, Nuerk, & Moeller, 2014; Slusser et al., 2013).

Third, in case of the non-symbolic estimations, we were the first to demonstrate, on the basis of both cross-sectional and longitudinal data, that most young children’s estimation patterns were best described by a logarithmic model (within the log-to-lin account), whereas the estimation patterns of most older children were best described by the simple power model (within the proportion judgment account). These results demonstrate a similar, but different development for non-symbolic and symbolic estimation patterns: For both types of stimuli, the proportion judgment account best reflected the estimation patterns of at least the older children, but whereas in the symbolic data the *n-*cycle power model was for most childen the “best” model in each of the model comparisons, in the non-symbolic data the simple power model appeared to be the “best” model for most children. Where being best described by the proportion judgment account for the symbolic number line data reflected children’s use of certain number line estimation strategies, this interpretation does not hold for the non-symbolic number line data. The observation that most non-symbolic estimation patterns are best described by the less advanced (i.e. simple power) model within this developmental account suggests that even older children do not seem to be able to employ particular anchor-based strategies when solving this task, although they know the begin- and endpoint value of the line (see instructions). Furthermore, in case of the youngest children, we observed that the logarithmic model described the estimation patterns better than the simple power model – although both models are very similar (see Footnote 1 and Figures 1A versus 1D). Most likely, the latter can be explained by young children’s tendency to overestimate small numbers (Meijas & Schiltz, 2013), which results in a logarithmic curve (see e.g. Figure 1A), while the more adequate estimations of small numbers from older children are better described by a simple power model (see e.g. Figure 1D).

Finally, it must be noted that not all children exhibited the expected evolution from the less advanced to the more advanced model in a particular account. In Table 6, for example, it can be observed that some children whose estimation pattern was best described by a *n*-cycle model in the first grade (T1), dropped back one year later, as reflected in a better description by the simple power model at T2. This finding has also been observed by other researchers: Slusser et al. (2013) for instance already reported that the cyclic models of older children sometimes showed a reversed pattern. However, the reason for such reversed patterns is not yet understood well. Future studies should therefore focus on further unravelling the individual estimation trajectories by examining the relationship between number line estimation performance and the performance on a variety of cognitive ability tests in order to reveal which cognitive abilities may play a role in the development of these kind of patterns.

## Conclusions

In sum, because of its use of one single criterion to compare the three developmental accounts (i.e. the AICc), the present study provides the strongest evidence to date that the development of children’s *symbolic and non-symbolic* number line estimations does not reflect a developmental change in their mental representations of number, but rather the extent to which they might be using strategies such as using (internal) anchor points. Whereas all elementary school children are successful in this with symbolic number lines, the application of such strategies appears still too difficult in case of non-symbolic number lines, even for the oldest ones. Investigating whether, and to what extent, adults might be able to apply these strategies on non-symbolic number lines could offer solace with respect to the research question whether the development of non-symbolic number line estimations is characterized by a similar developmental trajectory as for symbolic number line estimations – albeit with a delay – or whether a different mechanism underlies symbolic and non-symbolic number line estimations.

## Footnotes

The power model is able to fit the same data as a logarithmic model and both models have been used in psychophysical studies to model estimation data that do not have a one-to-one relationship with the to be the to be estimated physical stimulus (i.e. Weber-Fechner law).

An exponential function in the Position-to-Number (P-N) task is essentially a mirror image of the logarithmic function in the Number-to-Position task (N-P) (Ashcraft & Moore, 2012).

To improve comparability between the different developmental accounts, we collapsed children whose estimation pattern was best fit by either a one-cycle or a two-cycle power model into one category: *n*-cycle power model. This way, a similar dichotomy was achieved for each developmental account.
